# Fecal shedding of *Brachyspira* spp. on a farrow-to-finish swine farm with a clinical history of “*Brachyspira hampsonii*”-associated colitis

**DOI:** 10.1186/1746-6148-9-137

**Published:** 2013-07-11

**Authors:** Amy H Patterson, Joseph E Rubin, Champika Fernando, Matheus O Costa, John CS Harding, Janet E Hill

**Affiliations:** 1 Department of Veterinary Microbiology, University of Saskatchewan, 52 Campus Drive, Saskatoon, SK S7N 5B4, Canada; 2 Department of Large Animal Clinical Sciences, Western College of Veterinary Medicine, University of Saskatchewan, Saskatoon, SK, Canada

**Keywords:** *Brachyspira*, Swine, PCR, Culture, Surveillance, Shedding

## Abstract

**Background:**

*Brachyspira* associated diarrhea is a re-emerging concern for Canadian swine producers. To identify critical control points for reducing the impact of *Brachyspira* on production, improved diagnostic tools and a better understanding of the on-farm epidemiology of these pathogens are required. A cross-sectional study was conducted for the detection of *Brachyspira* on a commercial, two-site, farrow-to-finish pork production unit in Saskatchewan, Canada with a clinical history of mucohaemorrhagic colitis associated with “*B. hampsonii*”.

**Results:**

Rectal swabs from pigs at all production stages were collected over 13 weeks (n = 866). Two swabs were collected per pig for culture and Gram stain, and for PCR. Ninety-one culture positive samples were detected, with the highest prevalence of *Brachyspira* shedding in grower pigs (21%). No *Brachyspira* were detected in pre-weaned piglets. PCR and Gram stain of rectal swabs detected fewer positive samples than culture. The most prevalent species detected was *B. murdochii*; other species detected included *B. pilosicoli, B. innocens,* and “*Brachyspira hampsonii*”. Phylogenetic analysis revealed that several of the isolates, including some strongly beta-haemolytic isolates, might represent novel taxa.

**Conclusions:**

Our results indicate that apparently healthy pigs can be colonized with diverse *Brachyspira* species, including some potential pathogens, and that frequency of shedding peaks in the grower stage. Difference in the detection rates of *Brachyspira* amongst culture, Gram stain or PCR on rectal swabs have implications for choice of detection methods and surveillance approaches that may be most effective in *Brachyspira* control strategies.

## Background

Bloody diarrhea associated with *Brachyspira* has re-emerged as a significant concern for swine producers in Canada. Historically, mucohaemorrhagic colitis (swine dysentery) in pigs is associated with *Brachyspira hyodysenteriae*, although other species such as *B. pilosicoli* have been shown to cause less severe, non-bloody diarrhea (porcine intestinal spirochetosis) [1]. Since 2009, outbreaks of bloody diarrhea indistinguishable from swine dysentery have been documented in grow-finish pigs in western Canada [2], and there are similar reports from the United States of increasing numbers of colitis cases associated with *Brachyspira*, including “atypical” strains [3]. Investigation of these cases led to the recognition of novel, strongly beta-haemolytic *Brachyspira* isolates, for which the name “*Brachyspira hampsonii*” has been proposed [4]. Experimental inoculations of pigs have established the pathogenic potential of this new species [5,6]. The re-emergence of *Brachyspira* spp. including *B. hyodysenteriae* and novel species like “*B. hampsonii*” as pathogens of concern has re-ignited interest in disease management and eradication, but identification of critical control points for reducing the impact of *Brachyspira* on pork production systems have been hampered by inadequate diagnostic tools and a limited understanding of on-farm epidemiology in modern multiple-site farms.

Culture methods for *Brachyspira* were established with the initial characterization of *B. hyodysenteriae*[7], but these methods have been gradually displaced in many diagnostic laboratories by species-specific conventional and quantitative PCR assays for the detection of previously characterized pathogenic *Brachyspira* species. PCR methods offer a less labour-intensive approach than culture, rapid turnaround time, and are amenable to automation. However, an obvious drawback is that these methods may not detect novel species and may not be adequate to detect all strains within even established species, which can be highly variable [8]. Also, lack of standardization of techniques among laboratories complicates comparison of results. To reduce analytical specificity in situations where the target species are not known, culture or more broad-spectrum PCR approaches such as genus-specific PCR based on the NADH oxidase (*nox*) gene may be useful [9]. Analytical sensitivity is another critical parameter, especially for surveillance in healthy pigs where detection limit is a particular concern and ante-mortem samples are limited to feces or rectal swabs.

Its fecal-oral transmission route, combined with a variety of possible animal and environmental reservoirs provide ample opportunities for *Brachyspira* transmission either directly or indirectly between pigs and farms [10]. Thus, an understanding of shedding patterns throughout the production cycle is essential for development of effective intervention strategies. A particularly pressing question is regarding the prevalence of shedding by sows and gilts since this has implications for designing medication and eradication strategies, and making decisions about the relative emphasis of intervention in the breeding herd, versus the grow-finish population. The current cross-sectional study was designed with two major objectives: to determine shedding patterns of *Brachyspira* throughout the production stages on a farrow-to-finish farm with a history of mucohaemorrhagic colitis associated with “*B. hampsonii*”, and to evaluate the performance of three different detection strategies: selective culture, Gram stain, and PCR on rectal swab samples collected from pigs with normal feces. We found that *Brachyspira* are shed by pigs throughout the production cycle, with the exception of suckling piglets, and confirmed the value of selective culture from rectal swabs for surveillance. Our results also reveal a startling diversity of *Brachyspira* spp. isolated from healthy pigs.

## Results

### Fecal shedding through production stages

Ninety-one of 866 swabs were culture positive for *Brachyspira* (Table 1). During the initial stages of the study, dark field microscopy was performed on samples of growth from haemolytic zones to ensure that these corresponded to the presence of bacteria with morphologies consistent with *Brachyspira*, and thus that the selective culture media was performing as expected. Culture results on CVS and BJ were generally concordant although overgrowth of fecal microbiota was more frequent on CVS compared to the more selective BJ. Prevalence of shedding was highest in grower pigs (47/222, 21%) and *Brachyspira* was cultured from all production stages except for the suckling piglets. Grower pigs were more likely to shed any *Brachyspira* species than young (OR = 9.3, 95% confidence interval (CI) 1.7-51.5, P = 0.01) or old (OR = 6.8, 95% CI 1.4-33.1, P = 0.02) nursery pigs, or sows (OR = 2.0, 95% CI 1.0-5.0, P = 0.07). Prevalence of shedding was similar in finishers (9.3%), gilts (7.7%) and sows (8.9%), and all higher than young and old nursery pigs (2.6% each).

**Table 1:** **Prevalence of *Brachyspira *in rectal swabs from all production stages, determined by culture**

	**Site 1**			**Site 2**			
**Week**	**Gilts**	**Sows**	**Suckling piglets**	**Youngest nursery**	**Oldest nursery**	**Grower**	**Finisher**
A	3/21	0/12	0/6	0/6	1/6	7/24	0/12
B	3/21	2/12	0/6	0/6	0/6	10/24	1/12
C	2/21	0/12	0/6	1/6	1/6	5/24	0/12
D	0/21	4/12	0/6	0/6	0/6	3/24	1/12
E	1/21	0/12	0/6	0/6	0/6	2/24	0/12
F	4/21	3/12	0/6	0/6	0/6	9/24	4/12
G	2/21	0/12	0/6	0/6	0/6	2/24	2/12
H	0/21	0/12	0/6	1/6	0/6	2/24	1/12
I	0/10	2/12	nd^1^	0/6	0/6	nd	nd
J	1/10	1/12	nd	0/6	0/6	1/6	nd
K	0/10	1/12	nd	0/6	0/6	3/6	nd
L	1/10	1/12	nd	0/6	0/6	0/6	nd
M	0/10	0/12	nd	0/6	0/6	3/12	nd
Total	17/218 (7.7%)	14/156 (8.9%)	0/48 (0%)	2/78 (2.6%)	2/78 (2.6%)	47/222 (21%)	9/96 (9.3%)

^1^*nd*, not done.

The odds of *Brachyspira* shedding detected by Gram stained fecal smears was significantly higher in gilts (OR = 1.8, 95% CI 1.0-3.2, P = 0.045), sows (OR = 2.5, 95% CI 1.2-5.5, P = 0.02), growers (OR = 2.6, 95% CI 1.7-3.9, P < 0.01) and finishers (OR = 1.9, 95% CI 1.2-3.1, P < 0.01) than the oldest nursery pigs. The odds of Gram stain positives were also higher in sows (OR = 2.1, 95% CI 0.9-5.0, P = 0.09), growers (OR = 2.1, 95% CI 1.3-3.6, P < 0.01) and finishers (OR = 1.6, 95% CI 1.0-2.7, P = 0.07) than in young nursery pigs.

**Figure 1:**
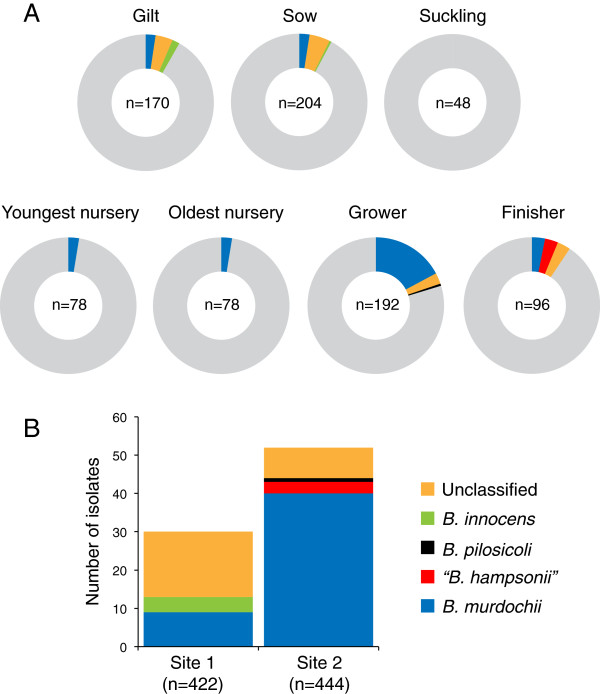
***Brachyspira *species detected by culture. (A)** Culture results for each production stage at Site 1 (upper tier) and Site 2 (lower tier). The total number of samples tested is indicated in the centre of each chart. Culture negative samples are grey. **(B)** Abundance of different *Brachyspira* species detected among culture positive samples at Site 1 and Site 2. Identification of isolates was based on partial *nox* gene sequence and phylogenetic analysis.

Partial *nox* sequencing was performed on 83 successfully sub-cultured isolates, and high quality sequence consistent with pure templates was obtained for 81 of these [Genbank: KC561373-KC561453]. Based on comparison of these sequences to available sequence data, four species were identified: *B. murdochii* (n = 49), *B. innocens* (n = 4), “*B. hampsonii*” (n = 3), and *B. pilosicoli* (n = 1) (Figure 1). Most *B. murdochii* isolates (32/49, 65.3%) were from grower pigs, and growers were significantly more likely to shed *B. murdochii* than young nursery (OR = 9.1, 95% CI 2.5-33.6, P < 0.01) and old nursery (OR = 10.2, 95% CI 2.8-37.8, P < 0.01), finishers (OR = 7.1, 95% CI 2.6-19.0, P < 0.01), gilts (OR = 7.9, 95% CI 3.9-18.9, P < 0.01) or sows (OR = 7.8, 95% CI 3.1-19.4, P < 0.01) (Figure 1A).

Almost one third (24/81, 30%) of isolates were ≤96% identical to any of the reference *nox* sequences, and did not cluster with reference sequences in the phylogenetic analysis (see below). Even though growers had the highest prevalence of shedding, sows tended to be more likely than growers to shed “unclassified” *Brachyspira* (OR = 3.6, 95% CI 0.84-16.1, P = 0.08) (Figure 1A).

### Phylogenetic analysis of cultured isolates

**Figure 2:**
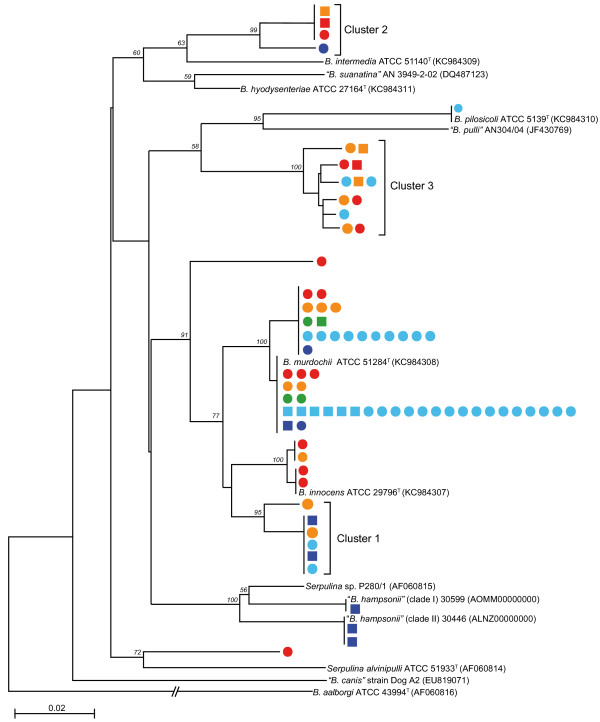
**Phylogenetic tree of partial *nox *gene sequences from 81 cultured *Brachyspira *isolates.** Representative strains of recognized species are included for reference. Numbers at the nodes indicate bootstrap values out of 100 iterations. Scale bar indicates number of substitutions per site. Production stage is indicated by colour (legend) and square markers indicate strongly beta-haemolytic isolates. Genbank accession numbers are indicated in parentheses for published sequences. Clusters of unclassified isolates (Clusters 1, 2 and 3) are indicated, and discussed in the text.

A phylogenetic analysis of the partial *nox* sequences determined for 81 study isolates is shown in Figure 2 (*nox* sequences of two isolates were too short for inclusion). Three clusters (Clusters 1, 2 and 3) and several single isolates grouped separately from any reference sequences with good bootstrap support. As expected, isolates corresponding to “*B. hampsonii*” strains 30599 and 30446 (corresponding to “clade I” and “clade II” of “*B. hampsonii*”, respectively [4]) were strongly beta-haemolytic, but strongly beta-haemolytic isolates were also detected among *B. murdochii*-like isolates (8/49 isolates), although most *B. murdochii* isolates (41/49) were weakly beta-haemolytic as expected based on the species definition. Strongly beta-haemolytic isolates were also identified among isolates in Cluster 1 (2/6 isolates), Cluster 2 (2/4 isolates) and Cluster 3 (3/12 isolates) (Figure 2). These strongly beta-haemolytic isolates were cultured from rectal swabs of all culture-positive age groups, but most (13/18, 72%) were from Site 2. Surprisingly few “*B. hampsonii*” isolates were found, and only in the finisher group at Site 2. *B. innocens* was detected only in gilts and sows at Site 1 (Figure 1). Other phylogenetic groups were identified in pigs from both production sites and a mixture of production stages.

### Environmental sampling

Environmental samples were collected within barns in weeks I-M (n = 198). Six of these samples (3%) from floor slats (n = 2), grow-finish manure pits (n = 3), and light fixtures (n = 1) were culture positive. Most of the positives (5/6) were identified as *B. murdochii*, with one pit isolate identified as *B. pilosicoli*.

Colon samples from all rats (n = 8) and mice (n = 5) trapped in weeks A, I and J were culture negative.

### Comparison of detection methods

**Figure 3:**
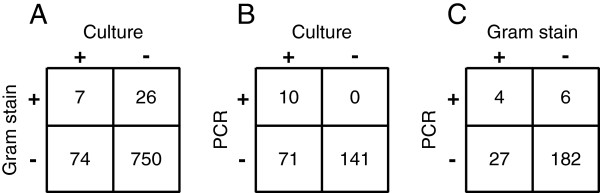
**Comparison of detection of Brachyspira by culture, PCR and Gram stain.** Contingency tables showing numbers of samples determined to be positive or negative for *Brachyspira* by culture, *nox* PCR on rectal swabs, and Gram stain of fecal smears. Results are shown for samples for which data was available for **(A)** Culture and Gram stain, **(B)** Culture and PCR, **(C)** Gram stain and PCR.

A total of 866 pigs were sampled over the 13-week study period, and examined by culture and Gram stain, and both results were successfully obtained for 857 samples. All Gram stain scores were ≤ 2+ (<10 spirochaetes/field). Of the 81 culture-positive samples, spirochetes were detected by Gram stain in only 7 samples (Figure 3A). A small number of samples (26/857, 3%) were culture negative but snake-like spirochaetes were visible on Gram stain.

To investigate the performance of direct genus-specific *nox* PCR on rectal swab extracts relative to culture, culture positive samples that had been successfully sub-cultured and identified by *nox* gene sequencing (n = 81) were matched with a set of culture negative samples. For each culture positive sample, at least one negative sample from the same week and production stage was selected. If no exact match was available, a negative sample from the next closest age group was selected. The result was a set of 222 samples for which both culture and dry swab *nox* PCR were evaluated (Figure 3B). All samples were positive for the internal control target, swine ND1. As observed for Gram stain, only a small proportion of culture positive samples were PCR positive (12%), and none of the culture negative samples were PCR positive. *nox* sequence was determined successfully for 6/10 culture and PCR positive samples and for four of these, the dry swab *nox* sequence was 99-100% identical to the *nox* sequence determined for the cultured isolate from the same pig. For pig F80 (23 week finisher, week F), the cultured isolate was 96% identical to *B. innocens* but the dry swab *nox* sequence was 96% identical to *B. murdochii*. For pig G79 (23 week finisher, week G), the dry swab nox sequence was also 96% identical to *B. murdochii*, while the isolate from this pig was 99% identical to “*B. hampsonii*” strain 30446. These results suggest dual infection in these two pigs.

We were able to compare detection by dry swab PCR and Gram stain for 219 samples and determined that the results agreed for 186/219 samples (Figure 3C).

## Discussion

Most studies of *Brachyspira* prevalence published to date have been based on targeted sampling of symptomatic pigs on farms with clinical diarrhea [11-17], while surveys including healthy pigs, and cross-sectional studies at the within-herd level are rare [18,19]. The focus on active outbreaks and cohort studies means that we lack basic information about the prevalence of *Brachyspira* in healthy pigs, and shedding patterns throughout production stages, both of which are critical for development of control strategies. Given the ongoing re-emergence of *Brachyspira* associated diarrhea as a production-limiting disease, information about *Brachyspira* prevalence, colonization and shedding in the North American production environment is certainly needed.

The farm we studied had a history of diarrhea associated with “*B. hampsonii*” but over the 13-week sampling period pigs were not medicated, and no clinical signs of dysentery were observed. Swine dysentery often exhibits a cyclical pattern at the farm level, with periodic appearance of clinical signs often after removal of therapeutic levels of antibiotics [20]; a pattern that suggests persistence of *Brachyspira* in pigs and/or their environment at a subclinical level. Not surprisingly given the farm history, “*B. hampsonii*” was detected in three finisher pigs on Site 2, however, its prevalence was lower than other species detected, and this species was not detected in grower pigs (Figures 2, 1A), where clinical signs were frequently observed in the two years prior to the study. The other presumed pathogen detected in the study was *B. pilosicoli*, detected in one grower pig and one environmental sample, also on Site 2. It is noteworthy that no *B. hyodysenteriae* was detected or isolated from the farm.

Taken together, the culture results showed the prevalence of fecal shedding of *Brachyspira* was higher on Site 2 than Site 1, and the distribution of species also differed between sites (Figure 1B). Swine dysentery is usually a disease of growing pigs (15–70 kg), but may occur in older pigs, and rarely in suckling piglets [20]. No *Brachyspira* was detected in any of the suckling piglets sampled in this study (n = 48) using any of the techniques employed. However, *B. innocens, B. murdochii* and unclassified *Brachyspira* were isolated from sows, indicating that suckling piglets are exposed to *Brachyspira* through sow feces but may not be colonized due to host factors such as maternal immunity, an immature colon mucosa, and a milk diet that results in an intestinal environment and microbial community structure that does not support *Brachyspira* survival. In the present study, earliest shedding was detected in a small number of young nursery piglets at Site 2.

The most frequently isolated species was *B. murdochii* (49 isolates). *B. murdochii* was originally identified as *Serpulina murdochii* from a collection of swine isolates in Australia [21]. Its pathogenicity is debated, but there are reports of compelling associations with clinical disease [22], and mild cattharal colitis has been observed in experimentally inoculated pigs [23]. One defining characteristic of *B. murdochii* is weak beta-haemolysis [21], so the observation of strong beta-haemolysis in 8/49 (16%) isolates identified as *B. murdochii* based on *nox* gene sequence was unexpected (Figure 2). The culture plates were read by an experienced observer, familiar with the strong and weak beta-haemolysis phenotypes of *Brachyspira* spp. such as *“B. hampsonii*” (strong) and *B. innocens* (weak). It is possible, but unlikely, that all of these isolates were mixtures of strains since all were sub-cultured three times, and also, there was no evidence of mixture in the raw sequence data obtained from *nox* PCR products from these cultures (i.e. double peaks, high background, typical of mixed template sequencing). Strong beta-haemolysis is a defining characteristic of *B. hyodysenteriae*, and has been associated with other virulent isolates [4,24]. All of the strongly beta-haemolytic *B. murdochii*-like isolates were from grower and finisher pigs at Site 2, and they were detected across multiple sampling weeks (A, C, D, F, G, J, L and M). Since all of these isolates were cultured under the same conditions, it is likely that the different haemolytic properties observed are due to an underlying genetic difference rather than a phenotypic characteristic associated with different culture conditions. The pathogenicity of any of these atypical *B. murdochii* isolates either alone or in combination with other strains is currently unknown. *B. murdochii* was not detected in the original clinical disease investigation of mucohaemorrhagic colitis on this farm, so whether it was part of the disease process during the previous outbreak is not known. Further genotypic and phenotypic work is currently underway to characterize these isolates in detail.

Phylogenetic analysis of partial *nox* sequences resulted in the identification of three clusters containing isolates that were distinct from any of the recognized *Brachyspira* species (Figure 2). The significance of these isolates or their pathogenic potential is currently unknown, but further investigation is warranted.

Observations of unclassified spirochaetes exhibiting both weak and strong beta-haemolysis have been made in other studies [16,17,25,26]. There is an important distinction between these “unclassified” isolates and “atypical” *Brachyspira*, which are isolates with affiliation to established species based on sequence similarity but are phenotypically inconsistent with the species definition, such as the strongly beta-haemolytic *B. murdochii*-like isolates in this study or the 50 atypical isolates described in a survey of diarrheic pigs in the UK [16]. Either category presents a diagnostic challenge, but the unclassified isolates are particularly problematic for PCR-based diagnostic strategies. The pathogenic potential of any individual strain can be investigated in experimental inoculation studies, but the possibility that they may be contributors to disease in mixed infections is more difficult to demonstrate.

Clearly, further work is needed to define the genotypic and phenotypic characteristics of *Brachyspira* that are responsible for pathogenicity, and that would provide the most clinically relevant diagnostic markers. An alternative view is expressed by Weissenböck et al. [22] who postulated that perhaps total *Brachyspira* load in an animal is more important than the presence or absence of any particular species or strain, and that “under certain as yet to be established circumstances all members of the genus *Brachyspira* have pathogenic potential, once they are allowed to multiply extensively” [22]. Resolution of this issue will require more comprehensive investigations of clinical cases, and knowledge of whether a diverse *Brachyspira* community, as reported here, is a common or rare event in other pig farms.

Recognition of disease potential, or evaluation of the effectiveness of disease eradication efforts in a production system depends upon detection of shedding of *Brachyspira*, and the success of surveillance in turn depends upon the sampling and detection methods used. In the current study we compared selective culture from rectal swabs transported to the lab in transport medium, genus-specific *nox* PCR on rectal swabs transported to the lab without transport medium, and Gram stain of fecal smears made from the dry swabs. These three approaches were chosen since they represent a range of logistical challenges and cost, from inexpensive and rapid (Gram stain), through complex and relatively expensive (culture). Our goal was not to evaluate clinical sensitivity and specificity, but rather to determine which method would be most practical for research and surveillance applications.

The results of *nox* PCR on DNA extracts from rectal swabs transported to the lab in dry tubes were disappointing, with only 12% (10/81) of culture positive pigs detected positive by this method. Although the genus-specific PCR assay we used in our study was not developed for application on clinical samples, we chose to implement it because it is used routinely in our laboratory and others for the detection and identification of *Brachyspira* spp. either in culture, or directly from fecal and tissue samples. Application of a genus-specific method is potentially more cost-effective than species-specific methods for surveillance work where the particular species that will be encountered are not known, and a broad perspective is desired. We chose to collect samples for PCR with dry swabs since transport medium can be inhibitory to PCR [27]. The presence of PCR inhibitors in feces is well known, but positive results for the internal PCR control (pig ND1 gene) in all samples investigated (n = 222) showed that PCR inhibition was not a major issue in our study. We can also rule out the failure of the genus-specific *nox* primers to detect the *Brachyspira* in those samples since *nox* PCR on cultured isolates was successful on all sub-cultured isolates. Inefficient DNA extraction from scarce *Brachyspira* cells in the swab samples, and inadequate detection limit of a PCR assay targeting a low G + C target (~27%) may be the best explanations for the apparently low detection rate of the PCR assay used in this study. Although PCR offers a rapid and efficient diagnostic tool appropriate for samples from clinical cases where target levels are high, the data presented here show that culture from rectal swabs shipped immediately to the lab in transport medium is a superior choice for detection of sub-clinical levels of *Brachyspira* in feces when compared to the PCR protocol we employed in this study. Interestingly, 26 samples were culture negative but Gram stain positive for spirochaetes with snake-like morphology typical of *Brachyspira*. These samples may contain either *Brachyspira* spp. that are not viable under the culture conditions used (perhaps highly susceptible to the antibiotics used in the selective media), or they may represent morphologically similar spirochaetes of other genera; further evidence of even more un-catalogued intestinal spirochaete diversity.

## Conclusions

Our results indicate that fecal shedding of *Brachyspira* can be detected from pigs throughout the production cycle, with the exception of suckling piglets, and that frequency of shedding peaks in the grower stage. We also demonstrated that selective culture followed by species identification based on genus-specific PCR and *nox* gene sequencing was the most effective method for detection of *Brachyspira* shedding in healthy pigs among those we compared. The detection of established species, atypical and unclassified isolates on a two-site farm with a clinical history of mucohaemorrhagic diarrhea associated with “*Brachyspira hampsonii*” infection is an indication of the complexities of *Brachyspira* ecology and an indication that there is much work to be done in determining the potential role of these diverse organisms, alone or in combination, in causing diarrhea and colitis.

## Methods

### Study site

A commercial, two-site, 1,200 sow farrow-to-finish farm in Saskatchewan with a known history of “*B. hampsonii*” associated colitis was selected for the study. The farm was located in an isolated area of the province, with sparse hog farm density. Site 1 (breeding and farrowing) was populated in the mid 1980’s and had since received replacement gilts and boars from a number of sources. Site 2 (nursery, grow and finisher) was located approximately 10 km northeast of Site 1, from which it received 21-day-old piglets. Replacement gilts were reared in Site 2, and transported back to Site 1 when approximately 5–6 months of age. All pigs were housed indoors. Drinking water was supplied by deep wells at each site; water used for washing was periodically supplied from a local freshwater lake. The nearest hog farm neighbour was located 6 km north of Site 2. Trucking between sites, and to and from slaughter was done internally by the producer.

In June 2009, bloody diarrhea was infrequently noted in 11–24 week old pigs in Site 2. The affected pigs had been vaccinated for PCV2 and were pulse medicated with 33 g/T tylosin (1 week of 3) from 9 to 15 weeks of age to aid in the control of diarrhea, thought to be caused by *Lawsonia intracellularis*. There was no significant change in mortality following the first observations of bloody diarrhea. Examination of fixed and fresh tissues submitted from affected pigs between June and October 2009 failed to elucidate an etiology. Histologic examination confirmed a catarrhal and superficial necrotizing colitis. Bacterial cultures of the colon were negative for *Salmonella* spp., as were species-specific PCR for B*. hyodysenteriae, B. pilosicoli* and *B. innocens*. Subsequent *Brachyspira* genus-specific PCR testing of archived samples, and newly submitted pigs with mucohaemorrhagic diarrhea detected “*B. hampsonii*” (strain 30446, clade II) in the absence of other known enteric pathogens including known pathogenic *Brachyspira* species. Pulse medication of grower pigs continued as above, whereas finisher pigs were medicated episodically only when clinical signs warranted. Mortality remained low (3.5-4.0% in combined nursery-grow-finisher) and over the course of 2 years (2010–2012) the severity and frequency of clinical signs gradually decreased. Feed medications were removed from all grow-finish diets in May 2012 at the initiation of this field study and remained out for the entire study and beyond.

### Sampling

**Figure 4:**
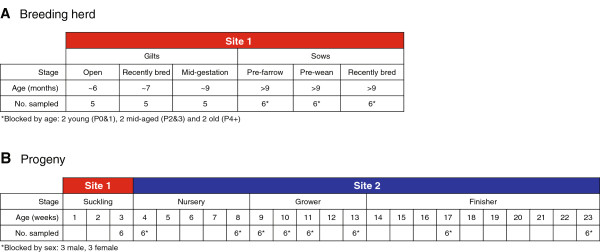
**Sampling strategy for the two-site farrow-to-finish farm.** Location (Site 1 or Site 2), production stage, pig age and sample number are shown for the breeding herd **(A)** and progeny **(B)**.

Samples were collected on each of 13 consecutive weeks (weeks A-M) over the summer of 2012 (Figure 4). For each of the first 8 weeks (A-H), 87 pigs were sampled. Weekly sampling at Site 1 included open gilts (n = 5), recently bred gilts (n = 5), gilts in mid-gestation (n = 5), recently bred sows (n = 6), sows pre-farrowing (n = 6), sows pre-wean (n = 6), and the oldest suckling piglets (n = 6). Sucking piglets were conveniently sampled from the litters of the sows that were sampled. Sampling of sows was blocked by parity: 2 young (parity 0&1), 2 mid-aged (parity 2&3), and 2 old (parity 4+). In Site 2, gilt and barrow pigs were housed in separate rooms, as was each week of production, so sampling procedures accounted for blocking by age and sex. Samples from Site 2 included the youngest nursery piglets (n = 6), the oldest nursery piglets (n = 6), grower pigs 1, 2, 3, and 5 weeks post-placement (n = 6 each), and finisher pigs 4 and 10 weeks post-placement (n = 6 each). Each week, pens of each age group and sex were chosen randomly from their respective rooms, and 3 pigs conveniently sampled within each selected pen. During weeks I-M, 34 pigs were sampled; sampling of grower and finisher pigs was discontinued and sampling continued as described above for sows, gilts, youngest and oldest nursery piglets. This sampling strategy enabled more targeted sampling of low prevalence age groups and freed up resources to enable environmental sampling.

Two rectal swabs were collected per pig at each sampling time. A sterile ‘wet’ swab (BD BBL Culture Swab plus Amies gel without charcoal, BD Canada, Mississauga, ON) was used for Gram stain and culture. A ‘dry’ swab (Starswab II specimen collection and transport system, Starplex Scientific, Etobicoke, ON) was used for PCR. All swabs were transported from the farm to the lab in a Styrofoam cooler containing ice packs to keep samples chilled, but not frozen. Upon arrival, wet swabs were immediately used to inoculate culture plates and make Gram stain slides. Dry swabs were frozen at -80°C until processing for PCR.

In weeks I-M, in addition to pig samples, a variety of environmental samples were taken (n = 198) using the sterile ‘wet’ swabs described above with the objective of determining if *Brachyspira* could be detected on non-pig surfaces. Sampling effort focused on fixtures (door handles, light fixtures, vents, air ducts), equipment (feeders, waterers), floor slats, and in-barn manure pits. Prior to sampling environmental surfaces, swabs were moistened by inserting them into their transport media. The swab head was then rolled across the surface of interest for five seconds. Manure pits under the slatted floors were sampled by dipping a 1-metre long wooden dowel into the slurry, moving it from side to side for 5 seconds, and then swabbing the feces covered end of the dowel with a ‘wet’ swab. Rats (n = 8) and mice (n = 5) trapped as part of routine rodent control in weeks A, I and J were dissected and swabs of colon mucosa were cultured as described above for pig rectal swabs.

The study was approved by the University of Saskatchewan’s Animal Research Ethics Board, and adhered to the Canadian Council on Animal Care guidelines for humane animal use (Protocol #20120028).

### Culture and identification of *Brachyspira* spp

Wet swabs were cultured within 6 hours of collection on two types of selective blood agar plates: CVS [7] and BJ [28], each containing 5% sheep blood. Swabs were streaked first on the CVS medium followed by inoculation of the more selective BJ medium. Plates were incubated at 42°C in anaerobic jars with anaerobic gas packs (AnaeroGen, Oxoid Company, Nepean, ON) and dry indicator strips (BD BBL, BD Canada, Mississauga, ON) and were inspected for zones of weak or strong beta-haemolysis indicative of *Brachyspira* growth after 48 hours, and 96 hours. Presumptive positive cultures were sub-cultured and incubated under the same conditions described above. All isolates were sub-cultured three times prior to identification.

Sub-cultured beta-haemolytic isolates were identified by amplification and sequencing of a 924 bp region of the NADH oxidase (nox) gene using a previously established protocol and genus-specific PCR primers for *Brachyspira*[9].

Type strains of *B. hyodysenteriae* (ATCC 27164), *B. innocens* (ATCC 29796), *B. intermedia* (ATCC 51140), *B. murdochii* (ATCC 51284), and *B. pilosicoli* (ATCC 51139) were obtained from the American Type Culture Collection and cultured as described above. Partial *nox* sequences were generated for these isolates for comparison to study isolates and other published sequences from Genbank.

### Fecal smears and Gram stain

Fecal smear slides were prepared from wet swabs immediately after inoculation of culture plates. Twenty high power fields were observed under oil immersion at 100× and scored (0: no spirochetes seen, 1+: ≤1 spirochete/field, 2+: 2–10 spirochetes/field, 3+: 11–49 spirochetes/high power field, 4+: >50 spirochetes/field).

### PCR detection of *Brachyspira* in rectal swabs

Dry swabs were thawed and DNA was extracted using a commercial kit (QIAmp DNA Stool Mini Kit, Qiagen, Toronto, ON). Two PCR reactions were performed on each sample. For verification of sample integrity, a 278 bp region of the pig mitochondrial NADH dehydrogenase subunit 1 (ND1) gene was amplified with primers JH0183 (5′-TCA TCG GGG CCC TAC GAG CA-3′) and JH0184 (5′-GGC GAA AGG TCC GGC TGC AT-3′). Primers were designed based on the annotated ND1 gene within the *Sus scrofa* mitochondrial genome sequence (GenBank Accession NC_000845). ND1 PCR reactions were incubated at 94°C for 3 min, followed by 40 cycles of [15 sec at 94°C, 15 sec at 52°C and 15 sec at 72°C] and a final extension for 5 min at 72°C.

For detection of *Brachyspira* in dry swab extracts, the *nox* PCR described above was used. PCR products from positive samples were purified (EZ-10 Spin Column PCR Products Purification Kit, Bio Basic Inc., Markham, ON) and sequenced using the amplification primers.

### Sequence data analysis and phylogenetic trees

Raw sequence data from forward and reverse primers was assembled and edited using PreGap4 and Gap4 [29]. Initial identification and classification of *nox* sequences was done by BLASTn [30] comparison of sequences to a custom database of *nox* gene sequences that includes all published *Brachyspira nox* gene sequences from Genbank as well as sequences from clinical isolates generated in our laboratory. For phylogenetic analysis, study sequences and relevant reference sequences were initially aligned with CLUSTALw [31] and the alignment was trimmed to a uniform length. Bootstrapping, distance matrix calculation (F84 method), neighbor-joining and consensus tree calculation were done with PHYLIP [32].

### Statistical analysis

General estimating equations (Stata v11, StataCorp, College Station, TX) were used to determine if the frequency *Brachyspira* shedding differed between stages of production, while controlling for week of collection. For this analysis, stage of production was categorized: young nursery (wk 4), old nursery (wk 8), grower (combined wks 9–11, 13), finisher (combined wks 17, 23), gilts (open, recently bred, mid-gestation), and sows (recently bred, pre-farrow, pre-wean sows of all parities). Pig sex was forced into the model regardless of its significance because males and females were housed in separate rooms. There were sufficient numbers of positive samples to allow pairwise comparisons for four biologically relevant outcomes: culture positive for any *Brachyspira*, culture positive for *B. murdochii*, culture positive for *Brachyspira* isolates that do not match characterized species based on *nox* gene sequence, and the presence of spirochaetes on Gram stained fecal smears. P values < 0.05 were considered statistically significant, whereas P values between 0.05 and 0.10 were considered trending towards significant.

## Competing interests

The authors declare that they have no competing interests.

## Authors’ contributions

AHP, JER, MOC collected and cultured the samples. AHP and CF performed PCR and collected DNA sequence data. AHP and JEH conducted phylogenetic analysis. JSCH performed statistical analysis. JER, JEH and JSCH conceived of the study. All authors contributed to writing and editing, and approved the final manuscript.
